# Design, Fabrication, and Mechanical Properties of T-700^TM^ Multiaxial-Warp-Knitting–Needled–C/SiC Composite and Pin

**DOI:** 10.3390/ma15062338

**Published:** 2022-03-21

**Authors:** Xiao Luo, Jiangyi He, Xiaochong Liu, Youliang Xu, Jian Li, Xiaojun Guo, Qianru Wang, Longbiao Li

**Affiliations:** 1 AECC (Aero Engine Corporation of China), Hunan Aviation Powerplant Research Institute, Zhuzhou 412000, China; luoxiaodut@163.com (X.L.); youliang_xu@163.com (Y.X.); lijiannpu@163.com (J.L.); gxj608@163.com (X.G.); 2 Xi’an Golden Mountain Ceramic Composites Company, Xi’an 710065, China; hejiangyi1986@163.com; 3 National Key Lab of Thermostructure Composite Materials, Northwestern Polytechnical University, Xi’an 710072, China; topcmc@126.com; 4 College of Civil Aviation, Nanjing University of Aeronautics and Astronautics, No. 29 Jiangjun Ave., Nanjing 211106, China

**Keywords:** C/SiC, pin, double-shear test, mechanical properties

## Abstract

In this paper, the 12k T-700^TM^ Multiaxial-Warp-Knitting–Needle (MWK–N) C/SiC composite and pin were designed and fabricated using the isothermal chemical vapor infiltration (ICVI) method. The composite’s microstructure and mechanical properties were examined by subjection to tensile and interlaminar shear tests. Three types of double-shear tests were conducted for C/SiC pins, including shear loading perpendicularly, along, and at 45° off-axial to the lamination. The fracture surface of the tensile and shear failure specimens was observed under scanning electronic microscope (SEM). The relationships between the composite’s microstructure, mechanical properties, and damage mechanisms were established. The composite’s average tensile strength was σ_uts_ = 68.3 MPa and the average interlaminar shear strength was τ_u_ = 38.7 MPa. For MWK–N–C/SiC pins, the double-shear strength was τ_u_ = 76.5 MPa, 99.7 MPa, and 79.6 MPa for test types I, II, and III, respectively. Compared with MWK–C/SiC pins, the double-shear strength of MWK–N–C/SiC pins all decreased, i.e., 26.7%, 50.8%, and 8% for test types I, II, and III, respectively. The MWK–N–C/SiC composite and pins possessed high interlaminar shear strength and double-shear strength, due to the needled fiber in the thickness direction, low porosity (10–15%), and high composite density (2.0 g/cm^3^).

## 1. Introduction

The C/SiC ceramic-matrix composites (CMCs) have excellent properties such as high-temperature resistance, thermal shock resistance, high strength, toughness, hardness, wear resistance, chemical stability, design tolerance, and low density and thermal expansion coefficients [1,2,3,4,5]. It can meet the requirements of long service life at a high temperature of 1650 °C and has broad application prospects in high thrust-to-weight ratio (TWR) aero engines, hypersonic ramjet engines, space shuttle thermal protection systems (TPS), and so on [6,7,8,9,10]. The HERMES, MSTP, ARD, GSTP, and FLPP programs of the European Space Agency (ESA) and the NASP, ISTP, Future-X, Hyper-X, and OTV programs of NASA have carried out relevant research and demonstration verification tests on C/SiC TPS. NASA used flaps, nose cones, and other components made of C/SiC composites in X-38, which reduced the weight of the X-38 thermal protection structure by 50% [11,12,13].

Three different techniques are currently used in an industrial scale for the production of C/SiC and C/C–SiC composites, i.e., chemical vapor infiltration (CVI), liquid polymer infiltration (LPI) or polymer infiltration and pyrolysis (PIP), and liquid silicon infiltration (LSI). Fiber orientation, dimensionality of the preform, and thermal treatment conditions are important parameters of influence on the performance of the final CMC product [10]. Due to the needs of inspection, disassembly, and maintenance, all parts of composite materials need to be connected. The connection of composite parts is of great significance for the design and application of CMCs. Common connection methods include adaptive robot ceramic joining technology [14,15,16], welding [17,18] and mechanical connection [19,20,21]. Mechanical connection refers to the connection of materials with fasteners, including bolts, rivets, and pins. Li et al. [22] fabricated the C/SiC nuts and bolts using the precursor infiltration and pyrolysis (PIP) process and analyzed the effect of fiber preforms, machining methods, and machining time on the mechanical properties of C/SiC nuts and bolts. Zhang et al. [23] fabricated the C/SiC z-pinned joint using the chemical vapor infiltration (CVI) method and analyzed the effect of porosity of the z-pin on the shear properties of a z-pinned joint. The critical porosity for the shear-controlled failure to bending-controlled fatigue was approximately 17.7%. Li et al. [24] investigated the microstructure and tensile behavior of C/SiC z-pinned joints. The average shear strength reaches τ_u_ = 157.7 MPa and the main failure mechanisms involved debonding of the lap interface and fiber shear-off within the pin. Around the hole, intact morphology, matrix crushing, and pin/hole debonding appeared. Liu et al. [25] performed numerical simulation and experimental validation of C/SiC riveting joints under tensile loading. A multiaxial-warp-knitting (MWK) structure possesses high tensile strength and elastic modulus, strong designability, and good shear resistance [26,27,28]. The in-plane mechanical properties of the MWK composite are better than that of the plain-woven composite [29]. To improve the interlaminar performance of the MWK composite, the needled fibers are incorporated in the Z direction. However, in the above-mentioned research, the mechanical properties of C/SiC composites or pins with the MWK and needled fiber (MWK–N–C/SiC) have not been investigated.

The objective of this paper is to fabricate the 12k T-700^TM^ MWK–N–C/SiC composite and pin using the isothermal chemical vapor infiltration (ICVI) method and perform microstructure and mechanical properties experiments (i.e., tensile, interlaminar shear, and double-shear) on the composite and pin. Three types of double-shear tests are conducted for C/SiC pins, including shear loading perpendicularly, along, and at 45^o^ off-axial to the lamination. The fracture surface of the tensile and shear failure specimens is observed under a scanning electronic microscope (SEM). The relationships between the composite microstructure, mechanical properties, and damage mechanisms are established. In Section 2, the fabrication method of the 12k T-700^TM^ MWK–N–C/SiC composite and pin and the mechanical test procures for the tensile, interlaminar shear, and double-shear are introduced. In Section 3, the experimental results for the tensile and interlaminar shear of the composite, and the double-shear tests for three different types, are provided.

## 2. Fabrication of 12k T-700^TM^ MWK–N–C/SiC Composite and Pin and Mechanical Experimental Procedure

In this section, the fabrication method for the 12k T-700^TM^ MWK–N–C/SiC composite and pin is illustrated and the mechanical test procedures for determining the tensile, interlaminar, and double-shear properties for the composite and pins are also provided.

### 2.1. Fabrication of C/SiC Composite and Pins

The 12k T-700^TM^ MWK–N–C/SiC composite and pins are fabricated using the isothermal chemical vapor infiltration (ICVI) method. The carbon fiber’s preform is multiaxial warp-knitted in 0°/±45°/90° with needled fibers in the Z direction. The fiber’s preform volume is 40–45%. The pyrolytic carbon (PyC) interphase is deposited on the surface of the T-700^TM^ carbon fibers through the chemical reaction in Equation (1), and the PyC thickness is 0.1–0.2 μm. The deposition temperature is 1000 °C with a pressure of 5 kPa, a flow rate of Ar of 500 mL/min, C_3_H_6_ of 30 mL/min, and a deposition duration of 20 h.

(1) $${2C}_{x}H_{y}\left(g\right)\to {2\mathrm{xC}(g)+\mathrm{yH}}_{2}\left(g\right)$$

After the deposition of the PyC interphase, the SiC matrix is deposited on the fiber’s preform with the PyC interphase through the chemical reaction in Equation (2):

(2) $${\mathrm{CH}}_{3}{\mathrm{SiCl}}_{3}(g)\;\overset{H_{2}}{⟶}\;\mathrm{SiC}(s)+3\mathrm{HCl}(g)$$

For the deposition of the SiC matrix, the fabrication temperature is 1000 °C; the pressure is 5 kPa; the flow rate of H_2_ is 100 mL/min; the mole mixture ratio between H_2_ and MTS is 10; and the deposition duration is 120 h. To increase the density of the SiC matrix, multiple CVI processes are conducted until the density of the C/SiC composite is above 2.0 g/cm^3^, and the porosity volume is 10–15%.

After the deposition of the SiC matrix, the surface of the C/SiC composite is coated with SiC to seal the open porosity, and the thickness of the SiC coating is 50–100 μm.

### 2.2. Mechanical Test Procedures

**Figure 1:**
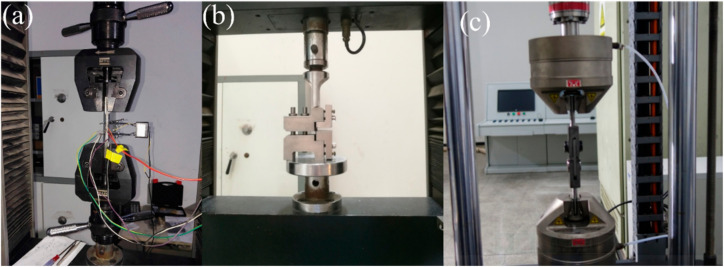
Mechanical tests of C/SiC composite and pins. (**a**) Tensile; (**b**) interlaminar shear; and (**c**) double-shear.

Figure 1a shows the monotonic tensile tests of the MWK–N–C/SiC composite. The tensile tests were conducted on an MTS CMT4304 testing machine (MTS Systems Corp., Minneapolis, MN, USA) following the ASTM-C1275 standard [30]. Tensile tests were under displacement control with a loading rate of 0.5 mm/min.

Figure 1b shows the interlaminar shear tests of the MWK–N–C/SiC composite. The interlaminar shear tests were conducted on an MTS CMT4304 testing machine (MTS Systems Corp., Minneapolis, MN, USA) following the ASTM-C1292 standard [31]. Interlaminar shear tests were under displacement control with a loading rate of 0.5 mm/min.

**Figure 2:**
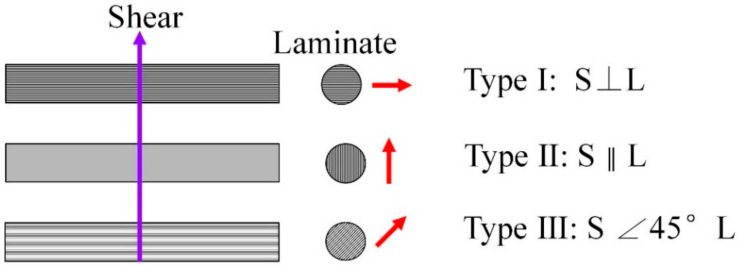
Schematic of three types of double-shear tests for the C/SiC pin.

Figure 1c shows the double-shear tests of the MWK–N–C/SiC pin. Double-shear tests were conducted on an Instron E10000 testing machine (Instron Company, Norwood, MA, USA) under the displacement control with a loading rate 0.5 mm/min. The diameter of the pin was approximately 4.2 mm, and the length of the pin was 30 mm. Three types of double-shear tests were conducted for the C/SiC pins (as shown in Figure 2), including:

- Type I, the shear loading is perpendicular to the lamination.
- Type II, the shear loading is along the lamination.
- Type III, 45° off-axial shear loading.

## 3. Experimental Results and Discussion

In this section, the tensile and interlaminar shear tests of the 12k T-700^TM^ MWK–N–C/SiC composite are conducted. The tensile damage behavior and tensile and interlaminar shear fracture strength are analyzed. For the 12k T-700^TM^ MWK–N–C/SiC pin, three double-shear tests are conducted. The double-shear fracture strength and related damage mechanisms are also discussed.

### 3.1. Tensile Behavior of 12k T-700^TM^ MWK–N–C/SiC Composite

**Figure 3:**
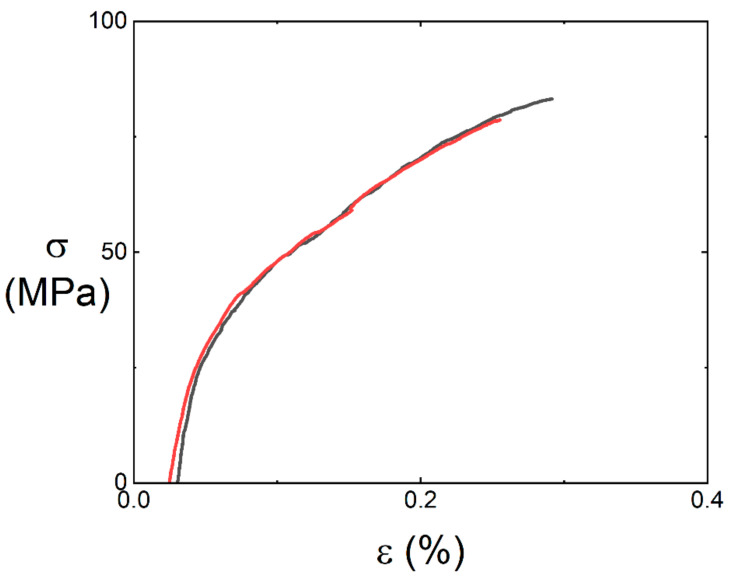
Monotonic tensile stress–strain curves of the 12k T-700^TM^ MWK–N–C/SiC composite.

Tensile behavior of the 12k T-700^TM^ MWK–N–C/SiC composite is investigated by subjection to tensile loading. After tensile fracture, the fracture surface of the sample is observed under a scanning electron microscope (SEM, FEI Company, Hillsboro, Ohio, USA) to show the tensile damage mechanisms. Figure 3 shows the tensile stress–strain curves of the 12k T-700^TM^ MWK–N–C/SiC composites at room temperature. Table 1 shows the tensile mechanical properties of the 12k T-700^TM^ MWK–N–C/SiC composites. Six samples were used to conduct the tensile experiments. The black and red curves in Figure 3 represent two samples for the tensile tests. The two samples’ tensile curves show obvious nonlinear behavior due to multiple damage mechanisms, e.g., matrix cracking, deflection of the cracks at the interface between the fiber and the matrix, and fiber fracture. The average composite tensile strength is σ_uts_ = 68.3 MPa and the average composite tensile modulus is *E* = 195.3 GPa.

**Figure 4:**
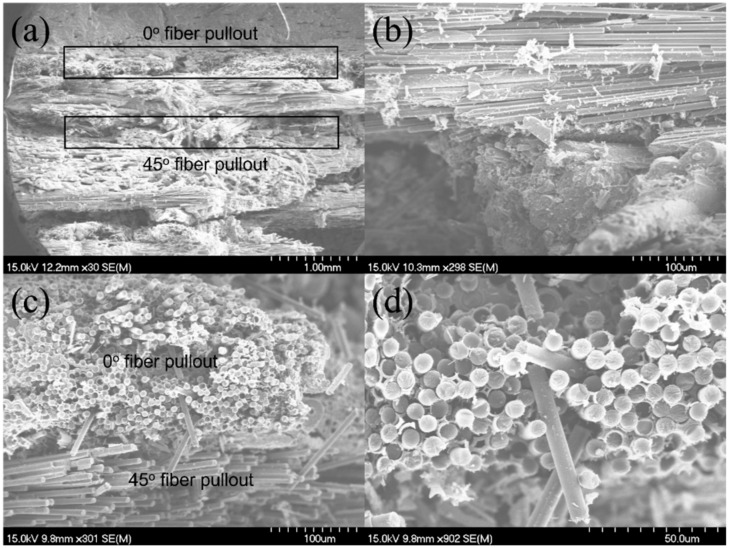
Tensile fracture surface of the 12k T-700^TM^ MWK–N–C/SiC specimen observed under SEM. (**a**) fibers pullout in the 0° and 45° plies; (**b**) fiber pullout in the 0° plies; (**c**) 0° and 45° fiber pullout; (**d**) fiber pullout in the 0° plies.

After the tensile fracture, the failure specimens were observed under the SEM, as shown in Figure 4. There exists 0°, ±45°, 90°, and needled fibers along the Z direction in the SiC matrix. From the observation of the fracture surface, it can be found that long fiber pullout exists in the 0° and ±45° fibers, as shown in Figure 4a,b; and the needled fibers along the Z direction appeared in the pullout fibers in the 0° and ±45° fibers, as shown in Figure 4c,d.

### 3.2. Interlaminar Shear Behavior of the 12k T-700^TM^ MWK–N–C/SiC Composite

Interlaminar shear behavior of the 12k T-700^TM^ MWK–N–C/SiC composite is investigated under the interlaminar shear test. After shear failure, the fracture surface of the samples is observed under the SEM. Table 2 lists the interlaminar shear properties of the 12k T-700^TM^ MWK–N–C/SiC composite at room temperature. Five specimens were conducted for the interlaminar shear mechanical tests. The average interlaminar shear strength is τ_u_ = 38.7 MPa.

**Figure 5:**
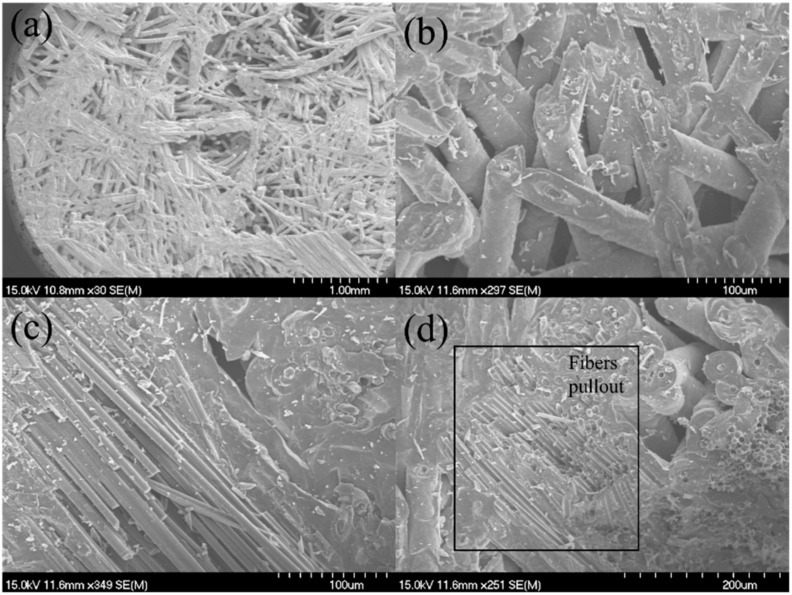
Interlaminar shear fracture surface of the 12k T-700^TM^ MWK–N–C/SiC specimen observed under SEM. (**a**) fracture surface of MWK–C/SiC specimen; (**b**) fracture fiber of MWK–C/SiC specimen; (**c**) needled fiber fracture in MWK–N–C/SiC specimen; (**d**) needled fiber pullout in MWK–N–C/SiC specimen.

After interlaminar shear failure, the fracture specimens were observed under the SEM, as shown in Figure 5. Compared with the MWK–C/SiC composite without needled fibers in the Z direction as shown in Figure 5a,b, needled fiber fractures and pullout in the Z direction appear, as shown in Figure 5c,d.

Under interlaminar shear loading, the needled fibers increased the energy dissipation along the crack path and improved the interlaminar shear strength. Needled fibers along the thickness direction can improve the resistance for the crack propagation between the laminar.

### 3.3. Double-Shear Mechanical Behavior of the 12k T-700^TM^ MWK–N–C/SiC Pins

**Figure 6:**
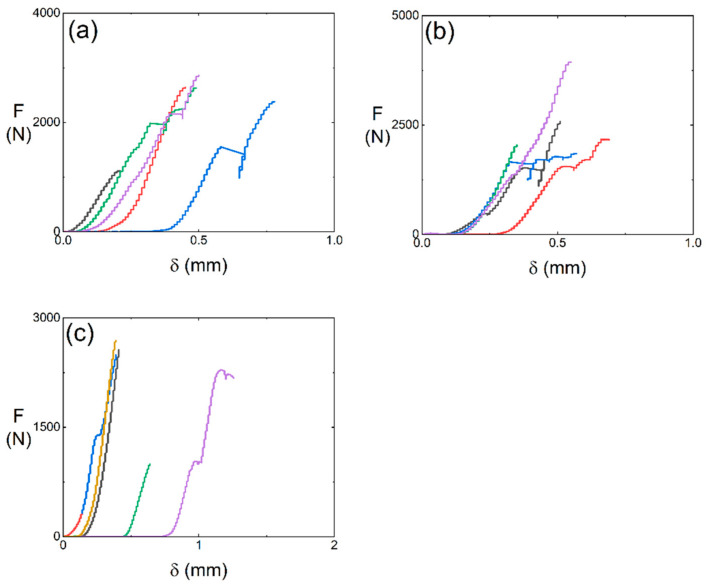
Double-shear load-displacement curves of the 12k T-700^TM^ MWK–N–C/SiC pins: (**a**) type I; (**b**) type II; and (**c**) type III.

The double-shear mechanical behavior of 12k T-700^TM^ MWK–N–C/SiC pins is investigated for test types I, II, and III. Comparison analysis of the double-shear strength among the three test types is also conducted. Figure 6 shows the load-displacement (*F*-*δ*) curves of the 12k T-700^TM^ MWK–N–C/SiC pins under double-shear loading for test types I, II, and III.

**Figure 7:**
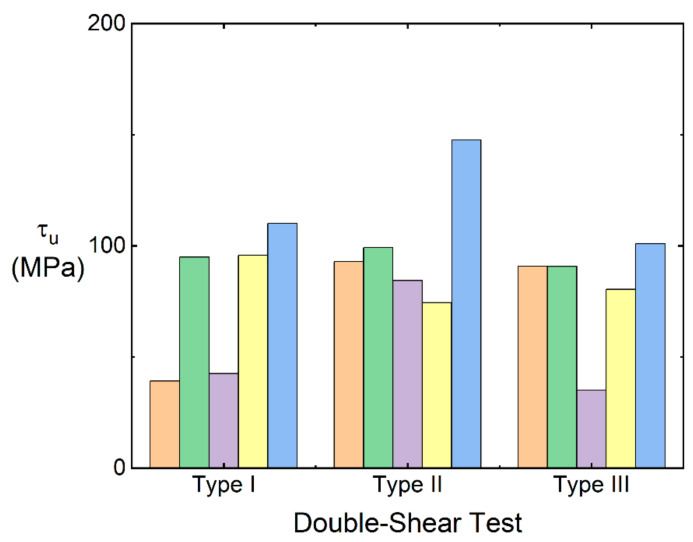
Double-shear strength of the 12k T-700^TM^ MWK–N–C/SiC pins.

Figure 7 shows the double-shear strength of the 12k T-700^TM^ MWK–N–C/SiC pins subjected to test types I, II, and III.

Table 3 lists the double-shear mechanical properties.

- For test type I, five samples (i.e., #1~#5) were conducted for the double-shear test. The average double-shear strength is τ_u_ = 76.5 MPa.
- For test type II, five samples (i.e., #6~#10) were conducted for the double-shear test. The average double-shear strength is τ_u_ = 99.7 MPa.
- For test type III, five samples (i.e., #11~#15) were conducted for the double-shear test. The average double-shear strength is τ_u_ = 79.6 MPa.

For the 12k T-700^TM^ MWK–C/SiC pins without needled fibers in the Z direction, the shear strength is τ_u_ = 104.3 MPa, 202.8 MPa, and 86.5 MPa for test types I, II, and III, respectively. Compared with the 12k T-700^TM^ MWK–C/SiC pins without needled fibers in the Z direction, the double-shear strength of the MWK–N–C/SiC pins all decreases in test types I, II, and III. By introducing the needled fibers in the C/SiC pins, the interlaminar fracture toughness can increase, however, the double-shear strength decreases 26.7%, 50.8%, and 8% for test types I, II, and III, respectively.

**Figure 8:**
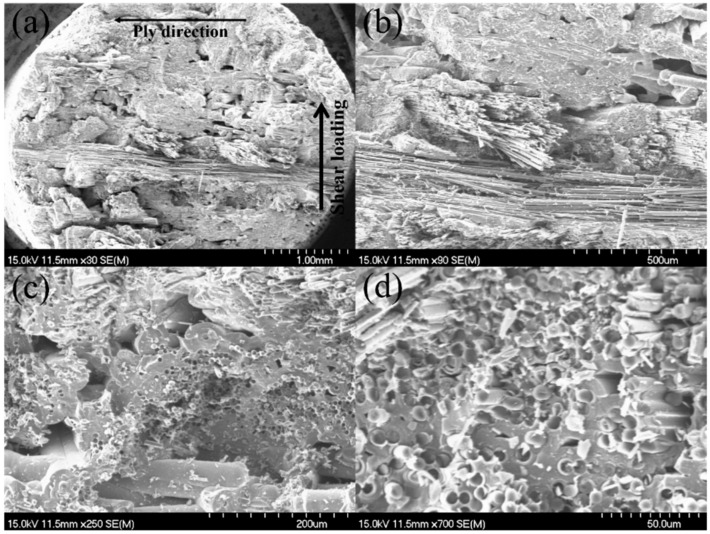
Type I double-shear fracture surface of the 12k T-700^TM^ MWK–N–C/SiC pin observed under SEM. (**a**) fracture surface; (**b**) fiber pullout in different plies; (**c**) fiber pullout; (**d**) fiber fracture and pullout.

**Figure 9:**
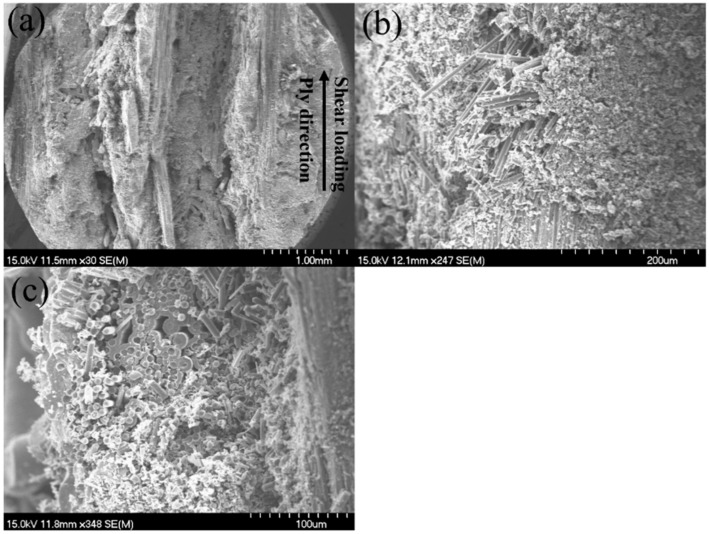
Type II double-shear fracture surface of the 12k T-700^TM^ MWK–N–C/SiC pin observed under SEM. (**a**) fracture surface; (**b**) fiber pullout in different plies; (**c**) fiber fracture and pullout.

**Figure 10:**
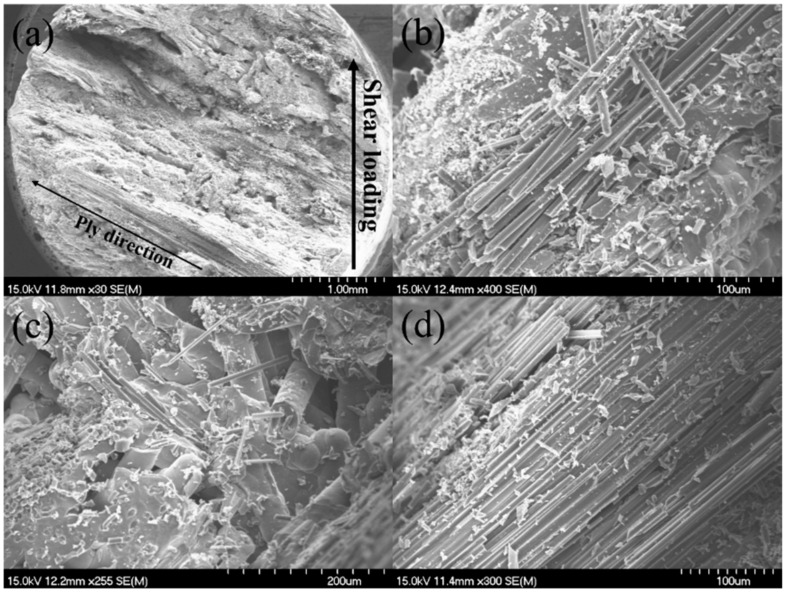
Type III double-shear fracture surface of the 12k T-700^TM^ MWK–N–C/SiC pin observed under SEM. (**a**) fracture surface; (**b**) fractured fibers; (**c**) fiber pullout; (**d**) fiber fracture and pullout.

Figure 8, Figure 9 and Figure 10 show the fracture surface of the 12k T-700^TM^ MWK–N–C/SiC pins for the double-shear test types I, II, and III. On the fracture surface, there exists obvious fiber pullout in the 0° and ±45° fibers, which increases the fracture toughness before shear failure. However, compared with the MWK–C/SiC pins, there is porosity and holes in the MWK–N–C/SiC pins. The needled fiber destroys the continuity of the in-plane fiber bundle and reduces the bearing capacity of the in-plane fiber. The shear strength of the pin in all directions is similar, which shows the significant effect of reducing the anisotropy of pins with needled fibers.

**Figure 11:**
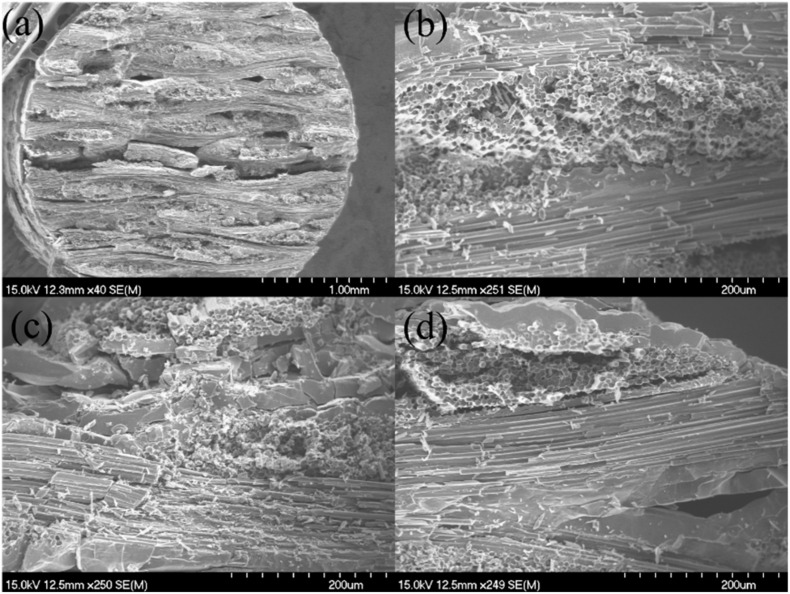
Type I double-shear fracture surface of 2D plain-woven C/SiC pin observed under SEM. (**a**) fracture surface; (**b**) fiber pullout in different plies; (**c**) fiber fracture and pullout; (**d**) fiber fracture and pullout.

Figure 11 shows the fracture surface of the 2D plain-woven C/SiC pin subjected to type I double-shear loading. Compared with the MWK–N–C/SiC pin, there are no needled fibers perpendicular to the lamination at the fracture surface, leading to the occurrence of the delamination between different plies.

### 3.4. Discussion

The mechanical properties of the C/SiC composites in the present analysis are compared with material data from other manufacturers. Table 4 lists the material data (e.g., tensile strength, Young’s modulus, and interlaminar shear strength, etc.) of the C/SiC composites fabricated using the CVI (isothermal), CVI (gradient), LPI, and LSI from NWPU, SNECMA, MAN, Dornier, MAN, and DLR. The tensile strength of MWK–N–C/SiC composite possessed low tensile strength compared with those C/SiC composites with (0°/90°) plain-woven (PW) fiber preform, due to the low fiber volume content along the tensile loading direction [32,33]. However, the Young’s modulus and interlaminar shear strength of the MWK–N–C/SiC composite were higher than those of the PW–C/SiC composite due to the needled fiber in the thickness direction, low porosity (10–15%), and high composite density (2.0 g/cm^3^). In fact, the needled fibers do not show high shear strength. On the other side, the decrease in the ceramic matrix porosity should have an exponential impact on the shear strength leading to a significant increase in its strength. For example, SICARBON™ materials produced by Airbus have a porosity higher than 20% and their interlaminar shear strength decreases from 15 MPa to 4 MPa when the porosity increases from 22 to 27% [34].

## 4. Summary and Conclusions

This paper fabricated 12k T-700^TM^ MWK–N–C/SiC composites and pins using the ICVI method and performed microstructure and mechanical properties experiments on the composites and pin. Three types of double-shear tests were conducted for the C/SiC pins, including the shear loading perpendicularly, along, and at 45° off-axial to the lamination. The fracture surface of the tensile and shear failure specimens was observed under SEM. Relationships between the composite microstructure, mechanical properties, and damage mechanisms were established.

- The average composite tensile strength was σ_uts_ = 68.3 MPa and the average composite tensile modulus was *E* = 195.3 GPa. At the fracture surface, long fiber pullout existed in the 0° and ±45° plies, and the needled fibers along the Z direction appeared in the pullout fibers in the 0° and ±45° fibers.
- The average interlaminar shear strength was τ_u_ = 38.7 MPa. Compared with the MWK–C/SiC composite, needled fiber fracture and pullout appeared in the Z direction.
- For the MWK–N–C/SiC pins, the double-shear strength was τ_u_ = 76.5 MPa, 99.7 MPa, and 79.6 MPa for test types I, II, and III, respectively. Compared with the MWK–C/SiC pins, the double-shear strength of the MWK–N–C/SiC pins all decreased, i.e., 26.7%, 50.8%, and 8% for test types I, II, and III, respectively.
- The tensile strength of the MWK–N–C/SiC composite possessed low tensile strength compared with those C/SiC composites with (0°/90°) plain-woven (PW) fiber preform, due to the low fiber volume content along the tensile loading direction. The Young’s modulus and interlaminar shear strength of the MWK–N–C/SiC composite were higher than those of the PW–C/SiC composite, due to the needled fiber in the thickness direction, low porosity (10–15%), and high composite density (2.0 g/cm^3^).

Temperature affects the mechanical behavior of the material [35,36]. In further studies, the mechanical properties of the MWK–N–C/SiC pins at elevated temperatures will be investigated to establish the relationship between the mechanical properties, temperature, load, and environment.

## Figures and Tables

**Table 1:** Tensile mechanical properties of the 12k T-700^TM^ MWK–N–C/SiC composite.

No.	Length/(mm)	Width/(mm)	Thickness/(mm)	σ_uts_/(MPa)	*E*/(GPa)
#1	130.2	11.6	3.2	82.8	161.9
#2	130.4	11.7	3.1	52.7	200.7
#3	130.2	11.6	3.1	57.5	154.2
#4	130.1	11.6	3.3	84.3	253.1
#5	130.2	11.6	3.2	54.1	277.4
#6	130.2	11.6	3.2	78.7	124.6

**Table 2:** Interlaminar shear properties of the 12k T-700^TM^ MWK–N–C/SiC composite.

No.	Width/(mm)	Notch Space/(mm)	Area/(mm^2^)	*F*_max_/(N)	τ_u_/(MPa)
#1	10.07	6.75	67.97	2522	37.1
#2	10.15	6.84	69.42	3254	46.8
#3	10.12	6.12	61.93	1616	26.1
#4	10.15	6.51	66.07	2773	41.9
#5	10.06	6.84	68.81	2882	41.8

**Table 3:** Double-shear mechanical properties of the 12k T-700^TM^ MWK–N–C/SiC pins.

No.	Diameter/(mm)	Density/(cm^3^)	Test Type	Ultimate Load/(N)	τ_u_/(MPa)
#1	4.24	2.44	I	1107	39.2
#2	4.21	2.42	I	2639	94.9
#3	4.15	2.47	I	1153	42.6
#4	4.17	2.47	I	2612	95.8
#5	4.04	2.47	I	2824	110.1
#6	4.17	2.48	II	2542	92.9
#7	3.73	2.51	II	2168	99.2
#8	3.73	2.51	II	1845	84.4
#9	4.16	2.56	II	2023	74.5
#10	4.11	2.49	II	3924	147.6
#11	4.21	2.52	III	2531	90.9
#12	4.17	2.49	III	2480	90.8
#13	4.17	2.53	III	958	35.1
#14	4.17	2.49	III	2199	80.5
#15	4.10	2.45	III	2668	100.9

**Table 4:** Overview of material data for the C/SiC composite.

Property	Unit	Gasphase Infiltration (CVI) Process			Liquid Infiltration Process		
		CVI (Isothermal)		CVI (p, T-Gradient)	Liquid Polymer Infiltration (LPI)		Liquid Silicon Infiltration (LSI)
		C/SiC	C/SiC	C/SiC	C/SiC	C/SiC	C/C–SiC
Tensile strength	MPa	52.7–84.3	350	300–320	250	240–270	80–190
Young’s modulus	GPa	124.6–253.1	90–100	90–100	65	60–80	50–70
Interlaminar shear strength	MPa	26.1–46.8	35	45–48	10	35	28–33
Porosity	%	10–15	10	10-15	10	15–20	2–5
Fiber content	Vol.%	30–45	45	42–47	46	42–47	55–65
Density	g/cm^3^	2.0	2.1	2.1–2.2	1.8	1.7–1.8	1.9–2.0
Fiber preform		MWK–N	(0°/90°) PW	(0°/90°) PW	(0°/90°) PW	(0°/90°) PW	(0°/90°) PW
Manufacturer		NWPU	SNECMA [10]	MAN [10]	Dornier [10]	MAN [10]	DLR [10]

## Data Availability

The data used to support the findings of this study are available from the paper.
